# Evaluating the effect of reference genome divergence on the analysis of empirical RADseq datasets

**DOI:** 10.1002/ece3.6483

**Published:** 2020-06-28

**Authors:** Justin Bohling

**Affiliations:** ^1^ Abernathy Fish Technology Center US Fish and Wildlife Service Longview WA USA

**Keywords:** biodiversity genomics, conservation genomics, restriction‐site associated DNA sequencing, Salmonidae, sequence alignment

## Abstract

The advent of high‐throughput sequencing (HTS) has made genomic‐level analyses feasible for nonmodel organisms. A critical step of many HTS pipelines involves aligning reads to a reference genome to identify variants. Despite recent initiatives, only a fraction of species has publically available reference genomes. Therefore, a common practice is to align reads to the genome of an organism related to the target species; however, this could affect read alignment and bias genotyping. In this study, I conducted an experiment using empirical RADseq datasets generated for two species of salmonids (Actinopterygii; Teleostei; Salmonidae) to address these questions. There are currently reference genomes for six salmonids of varying phylogenetic distance. I aligned the RADseq data to all six genomes and identified variants with several different genotypers, which were then fed into population genetic analyses. Increasing phylogenetic distance between target species and reference genome reduced the proportion of reads that successfully aligned and mapping quality. Reference genome also influenced the number of SNPs that were generated and depth at those SNPs, although the affect varied by genotyper. Inferences of population structure were mixed: increasing reference genome divergence reduced estimates of differentiation but similar patterns of population relationships were found across scenarios. These findings reveal how the choice of reference genome can influence the output of bioinformatic pipelines. It also emphasizes the need to identify best practices and guidelines for the burgeoning field of biodiversity genomics.

## Graphical abstract

A common practice in biodiversity genomics is to align high‐throughput sequencing reads generated for one species to the reference genome of a related species. In this study I aligned reads from empirical RADseq datasets generated for two species of salmonids to published reference genomes for six salmonid species of varying phylogenetic distance. Increasing phylogenetic distance between target species and reference genome reduced mapping success and quality, altered variant detection, and impacted inferences of population structure. These findings reveal that choice of reference genome is an important variable that affects the output of bioinformatic pipelines.

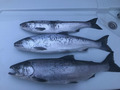

## 1 INTRODUCTION

High‐throughput sequencing (HTS) technologies have transformed the biological sciences, allowing biologists to generate prodigious amounts of data at genomic‐level scales. Methodological and bioinformatic advancements have opened HTS applications to nonmodel organisms, which have been a boon to fields such as conservation genetics, molecular ecology, and evolutionary biology (Allendorf, Hohenlohe, & Luikart, 2010; Ekblom & Galindo, 2011; Ellegren, 2014; Twyford & Ennos, 2012). Many of the bioinformatic approaches developed for HTS data require the use of established genomic resources as part of the pipeline. By “genomic resources,” I am referring to highly vetted consensus DNA sequences, whether at the whole genome, transcriptome, individual chromosome, or organelle‐level, that are meant to serve as a “reference” sequence for a given taxa (see Fuentes‐Pardo & Ruzzante, 2017 for a review). A reference genome provides a backbone on which to align HTS data and identify variants based on discrepancies between observed nucleotide base calls and the reference (Davey et al., 2011; Fuentes‐Pardo & Ruzzante, 2017; Nielsen, Paul, Albrechtsen, & Song, 2011).

It has reached a point where generating HTS data is not a significant barrier for geneticists in terms of cost and equipment availability (Fuentes‐Pardo & Ruzzante, 2017; Puckett, 2017). This has led to a torrent of HTS data for species across the Tree of Life. However, creating a quality reference genome is still a substantial obstacle that requires intense bioinformatic skill and financial investment (Ekblom & Wolf, 2014). Thus, HTS data are being generated for species that do not have a reference genome. A common practice is to use the reference genome of a species related to the target species of interest to align HTS data (Fuentes‐Pardo & Ruzzante, 2017). Published “whole‐genome comparisons” between multiple species often use the reference genome for one or a few of the species and then align whole‐genome resequencing data to them, opposed to actually generating a reference genome for each species (e.g., Árnason, Lammers, Kumar, Nilsson, & Janke, 2018; Cho et al., 2013; VonHoldt et al., 2016).

Aligning HTS reads from one species to the reference genome of another is not without peril. There should be greater sequence variability between species than within species, which could produce mismatches between the reference sequence and HTS reads and contribute to lower mapping quality in cross‐species alignments. Reads are likely to be mapped mainly to conserved regions between the species, inhibiting the detection of unique genomic variation within the target species (Fuentes‐Pardo & Ruzzante, 2017). Structural variants, repetitive elements, chromosome number, and copy number variation can also differ between species, affecting mapping and identification of paralogous genomic regions. Increasing divergence between the target species and reference genome can increase the number of missing genotype calls and produce biased estimates of population genetic parameters (Nevado, Ramos‐Onsins, & Perez‐Enciso, 2014; Shafer et al., 2017).

Although common practice, it is important to assess the efficacy of aligning HTS reads across species on both data quality and downstream interpretation. There have been several studies that have investigated its consequences. Nevado et al. (2014) simulated HTS datasets and reference genomes to assess the impact of genome divergence and sequencing coverage on estimation of population genetic parameters, observing a noticeable impact caused by reference genome divergence. Shafer et al. (2017) tested an empirical RADseq dataset generated for a species of pinniped (Mammalia; Carnivora; Pinnipedia) against a draft genome of the target species and reference genomes for three related species of increasing phylogenetic divergence. They also found reference genome divergence impacted population genetic parameters, but different metrics were more sensitive to divergence level. Gopalakrishnan et al. (2017) published a reference genome for the gray wolf (*Canis lupus*) and compared population genetic inferences for wild canids (Mammalia; Carnivora; Canidae) using their wolf genome and the commonly used domestic dog (*C. l. familiaris*) genome. Despite wolves and dogs having diverged within the last 30,000 years, the authors observed noticeable differences in some of the inferences for wild canids when the wolf genome was used.

These studies shed light on how divergence between reference genome and target species can impact the analysis of a HTS dataset. However, further research is still needed to assess the impact of such practices under different scenarios with empirical datasets, especially when making biological inferences, and the threshold at which genome divergence begins to have an effect. In this study, I assessed the impact of aligning HTS reads generated from two empirical RADseq datasets to real reference genomes of multiple species within the same taxonomic family. The RADseq datasets were for two species of salmonid (Actinopterygii; Teleostei; Salmonidae): Coho salmon (*Oncorhynchus kisutch*) and bull trout (*Salvelinus confluentus*). I aligned these data to publicly available reference genomes for six members of Salmonidae have been recently published, which is more than have been tested by other studies (Gopalakrishnan et al., 2017; Nevado et al., 2014; Shafer et al., 2017). They also provide a greater range of genomic divergence between target species and reference genome than has been previously tested. A reference genome is available for Coho salmon (Rondeau et al., 2017), allowing for a direct comparison of processing the RADseq data with the reference genome of the target species and species of increasing phylogenetic distance. No reference genome is available for bull trout but there is a genome for a closely related species within the genus, so performing a similar comparison provides an informative complement.

My objective was to evaluate the impact of reference genome divergence on mapping quality, number of variants, and inference of population structure. I also performed a de novo assembly of both RADseq datasets to assess whether coverage and inference were improved using contigs derived from the libraries themselves compared to aligning to a reference genome (Paris, Stevens, & Catchen, 2017). My hypothesis was that increasing phylogenetic distance between the target species and reference genome would result in fewer reads aligning and lower mapping quality, which would impact the discovery of variants. However, I anticipated that even if fewer variants were produced when using a more divergent reference genome, there would still be sufficient statistical power in the resulting data to reach the same biological interpretation (e.g., genetic differentiation) compared to using the genome of the target species. By utilizing recently developed genomic resources, this study provides insights into the practice of analyzing HTS data with the genome of another species.

## 2 MATERIALS AND METHODS

### 2.1 The genomes

**FIGURE 1:**
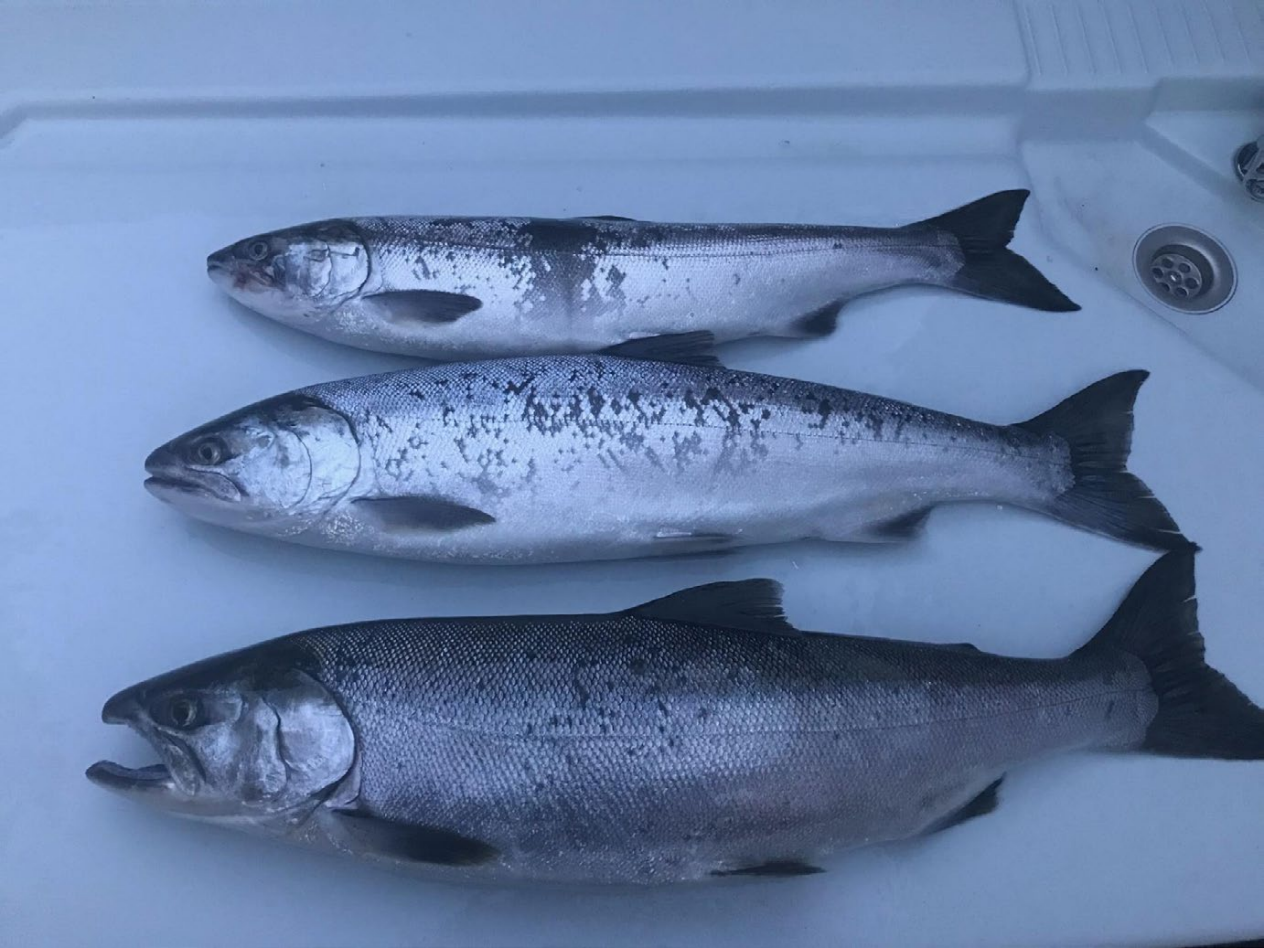
Photograph of two Coho salmon (top and middle fish) and Chinook salmon (bottom fish) harvested from the Columbia River. These are sister species within the genus *Oncorhynchus*. Photo courtesy of Steve Money

I used published whole genomes from six species within the family Salmonidae that represent a gradient of phylogenetic distance (Table 1). Among these available genomes, the two most closely related species are Coho (Rondeau et al., 2017) and Chinook salmon (*O. tshawytscha*) (Christensen, Leong, et al., 2018), which are sister species that diverged within the last ten million years (Figure 1) (Crête‐Lafrenière, Weir, & Bernatchez, 2012; Shedko, Miroshnichenko, & Nemkova, 2012). Within the same genus, there is a reference genome for the rainbow trout (*O. mykiss*) (Lien et al., 2017), which is part of a clade sister to the Pacific salmons (the group that includes Coho and Chinook salmon). A reference genome is available for the Arctic char (*Salvelinus alpinus*) (Christensen, Rondeau, et al., 2018): the genus *Salvelinus*, which also contains bull trout, is sister to *Oncorhynchus*, having split around 20–25 million years ago (MYA) (Crête‐Lafrenière et al., 2012; Lecaudey et al., 2018). Further diverged is the genome of the Atlantic salmon (*Salmo salar*) (Davidson et al., 2010; Lien et al., 2016): the genus *Salmo* is part of a clade that shares a most recent common ancestor with the *Oncorhynchus*/*Salvelinus* complex around 30–35 MYA (Crête‐Lafrenière et al., 2012; Lecaudey et al., 2018). All five of these species (Coho salmon, Chinook salmon, rainbow trout, Arctic char, and Atlantic salmon) are members of the subfamily Salmoninae. Basal to this subfamily is the group containing the European grayling (*Thymallus thymasllus*), which diverged around 60 MYA and also has a publically available reference genome (Varadharajan et al., 2017).

**TABLE 1:** List of the salmonid reference genomes used for this study. For the European grayling, the version of the reference genome used for this study was a draft assembly accompanying a preprint article (see text). The genome has subsequently been submitted to GenBank but after the completion of most of the bioinformatic analyses

Species	Common name	Year published	GenBank assembly accession	Assembly level
*Oncorhynchus kitush*	Coho salmon	2017	GCA_002021735.1	Chromosome
*Oncorhynchus tshawytscha*	Chinook salmon	2018	GCA_002872995.1	Chromosome
*Oncorhynchus mykiss*	Rainbow trout	2017	GCA_002163495.1	Chromosome
*Salvelinus alpinus*	Arctic char	2018	GCA_002910315.1	Chromosome
*Salmo salar*	Atlantic salmon	2015	GCA_000233375.4	Chromosome
*Thymallus thymallus*	European grayling	2017	NA	Scaffold

These six genomes represent varying levels of divergence from the two target species I included in this study. To quantity this divergence, I analyzed the annotated protein sequences associated with the reference genome assemblies for the six species using the OrthoFinder pipeline (Emms & Kelly, 2019). OrthoFinder identifies protein sequences that form orthogroups between species and then generates multiple gene alignments based on these groupings. I used the MAFFT (Katoh & Standley, 2013) option to construct the alignments and FastME 2.0 (Lefort, Desper, & Gascuel, 2015) to generate a neighboring‐joining tree based on genetic distance.

### 2.2 The datasets

The Coho salmon RADseq libraries were composed of samples collected from three populations (Bohling, Von Bargen, & Bahls, 2020). Two of these were from the Hood Canal region of Washington State, USA: one composed of hatchery broodstock propagated at Quilcene National Fish Hatchery (NFH) and another composed of spawning adults returning to Tarboo Creek, a nearby natural tributary. The third population consisted of wild‐origin Coho salmon from Warm Springs River in Oregon, USA, which is part of the Columbia River Basin. The Quilcene NFH broodstock was founded from local Hood Canal populations and Coho salmon from this region are highly diverged from Columbia River populations (Campbell & Narum, 2011; Van Doornik, Teel, Kuligowski, Morgan, & Casillas, 2007), meaning the samples used for this study should display a clear pattern of genetic structure. These three groups (Tarboo Creek, Quilcene, and Warm Springs) had similar sample sizes (*n* = 11–18) and produced comparable numbers of reads per individual (means of 1.96, 2.16, and 1.56 million reads, respectively; all individuals except one produced >500,000 reads).

The bull trout libraries contained samples from four populations (Bohling et al., 2019). Across its range in the United States, bull trout display several hierarchical phylogenetic aggregations. The main two lineages are the Coastal and Interior clades, which are separated by the Cascade mountain range (Ardren et al., 2011; Bohling et al., 2019; Spruell, Hemmingsen, Howell, Kanda, & Allendorf, 2003; Taylor, Pollard, & Louie, 1999). I selected data from two populations representing two distinct subclades within the Interior clade: the upper Columbia River (represented by Warm Spring Creek) and the Snake River (represented by Malheur River). For the Coastal clade, I selected populations representing two subclades: the Puget Sound (represented by Ruby Creek) and lower Columbia River (represented by the Lewis River). For the sequence data, these four populations had similar sample sizes (*n* = 15–16) and similar numbers of reads per individual (means of 2.0, 1.74, 1.6, and 1.42 million reads; no individual < 1,000,000 reads).

Procedures for generating the Coho and bull trout libraries were similar. To summarize, whole genomic data were digested with the *Sbf1* enzyme and then prepared following the original RADseq protocol of Baird et al. (2008). The Coho libraries were sequenced on an Illumina NextSeq500 for 100 forward cycles and 50 reverse; the bull trout libraries were sequenced on an Illumina HiSeq 2500 for 150 forward and reverse cycles. Each sample was indexed using a six base pair inline barcode and libraries demultiplexed using the *process_radtags* function in Stacks 2.0 (Catchen, Hohenlohe, Bassham, Amores, & Cresko, 2013). PCR duplicates were removed using the *clone_filter* function. Low‐quality reads, bases, and adaptor sequences were removed using Trimmomatic (Bolger, Lohse, & Usadel, 2014) as implemented in the dDocent pipeline (Puritz, Hollenbeck, & Gold, 2014) using the default parameters.

### 2.3 The sequence alignment and genotyping

I aligned the RADseq reads to each of the six reference genomes using Bowtie2 (Langmead & Salzberg, 2012) with the “—very‐sensitive” preset option for alignment sensitivity (the “—very‐fast” option was initially tested as well and produced similar alignment patterns in relation to reference genome choice). Other parameters were set to their defaults. To assess mapping quality associated with reference genome choice, I recorded mapping statistics generated from the *flagstats* command with Samtools (Li et al., 2009). I calculated mean MapQ after removing reads that did not map or had a MapQ < 1.

Nevado et al. (2014) found that certain genotyping software was more sensitive to genome divergence and sequence coverage. Therefore, I tested several different genotypers that have different analytical frameworks. Each program used the BAM files generated from Bowtie2 as input data. The first was Stacks 2.0 (Catchen et al., 2013), which uses a maximum‐likelihood approach to genotype variants based on with‐individual read distributions. This program is commonly used for RADseq data, and I used the SNP model for genotyping, which is the legacy model that has been part of earlier versions of the software. I also generated genotypes with FreeBayes (Garrison & Marth, 2012), which detects variants using a Bayesian approach that incorporates read distributions across all individuals. The final program was ANGSD (Korneliussen, Albrechtsen, & Nielsen, 2014), which can generate genotype probabilities based on overall read distributions and actual genotype calls. Within ANGSD, I used the GATK model for genotyping.

In comparing genotypes, I focused solely on biallelic SNPs as those are the only variants identified by ANGSD and Stacks SNP model does not produce indels, providing a fair comparison between the genotypers. For all SNPs identified by FreeBayes and Stacks, I calculated mean individual read depth at those sites using VCFtools (Danecek et al., 2011). Due to differences in output, read depth at SNPs identified by ANGSD could not be calculated. Default parameters were used in all applications.

### 2.4 The de novo assembly

For the Coho salmon and bull trout datasets, I performed a de novo contig assembly and subsequent variant identification. I processed the data through two popular pipelines: Stacks 2.2 (Catchen et al., 2013) and dDocent 2.6 (Puritz et al., 2014). Each pipeline used the reads remaining after the *clone_filter* procedure described above; I performed no filtering prior to running each pipeline. I used the default parameters for both pipelines and extended contigs using the reverse reads. Note that dDocent uses FreeBayes to call SNPs as part of its pipeline, making it comparable to use of FreeBayes with the reference‐aligned data. As with the reference‐based alignments, I focused only on biallelic SNPs and calculated mean individual read depth.

### 2.5 The population genetics

Because of the infinite possible ways to filter a HTS dataset, I tested a single potential scenario to focus primarily on the effect of reference genome on population genetic inference. In their simulations, Nevado et al. (2014) observed less variability in estimates of neutrality across genotypers with a read depth of 8X to retain genotypes. They also used 8X as their genotype threshold in their empirical case study, so I used 8X for the reference‐aligned and de novo datasets. I set a minor allele frequency threshold of 0.05 and only retained SNPs that were genotyped in more than 50% of individuals. To provide a fair comparison among the genotypers, I used called genotypes instead of genotype probabilities from ANGSD using the above filtering thresholds. Processing genotype probabilities requires specialized software with different algorithms and assumptions, which would prevent a direct comparison with the other two methods.

I performed two analyses to assess the impact of reference genome divergence on population genetic inference. First, I estimated overall *F*_ST_ (Weir & Cockerham, 1984) between populations for both species under the different scenarios using the package *assigner* (Gosselin, Anderson, Bradbury, 2016) for the R 3.6.1 statistical environment (R Core Team, 2018). I also performed a clustering analysis using Admixture (Alexander & Lange, 2011) to assess genetic structure. For each genome/genotyper combination, I used the cross‐validation approach to estimate the optimal number of genetic clusters in the dataset. I allowed the number of clusters (*K*) to vary from one to ten.

## 3 RESULTS

### 3.1 Reference genomes

**FIGURE 2:**
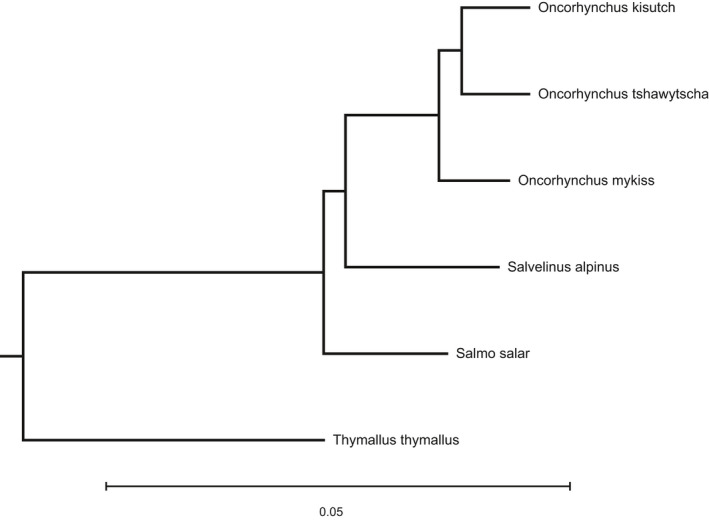
Neighbor‐joining tree based on genetic distances between the salmonid reference genomes used for this study. The tree was constructed using orthologous protein sequences identified by the OrthoFinder pipeline. It is a rooted tree, and branch lengths represent genetic distance. A scale bar depicting genetic distance is provided

The OrthoFinder pipeline recovered a phylogeny similar to that reported in other studies (Figure 2) (Crête‐Lafrenière et al., 2012; Lecaudey et al., 2018). Compared to the Coho salmon genome, both species within the genus *Oncorhynchus* (Chinook salmon and rainbow trout) had a sequence divergence between 1% and 2% (Table 2). The genome from other species in the subfamily Salmoninae (Arctic char and Atlantic salmon) was ~3.5% diverged from the Coho salmon genome. One unexpected result: although the phylogeny generated with the OrthoFinder pipeline (Figure 2) and those estimated by other studies (Crête‐Lafrenière et al., 2012; Lecaudey et al., 2018) place the genus *Salvelinus* sister to *Oncorhynchus* with *Salmo* as an outgroup, the actual estimated genomic divergence to the Coho salmon was slightly less for the Atlantic salmon genome compared to the Arctic char (Table 2). The European grayling genome was by far the most diverged at 8.7%. These divergence percentages are compared to those tested by Nevado et al. (2014) and Shafer et al. (2017) in Table 2.

**TABLE 2:** Summary of genomic divergence between target and related species tested by several studies. Genomic divergence is presented as the percent divergence between the species for which reference genomes were tested species and the target species for which the raw sequence data were generated. For bull trout (*Salvelinus confluentus*), there is no reference genome to calculate sequence divergence, so it was calculated from the Arctic char genome (*S. alpinus*) since they are from the same genus. Note that Nevado et al. (2014) simulated reference genomes of varying divergence and raw sequence data, along with testing *Gorilla gorilla* sequence data against a human assembly for a single chromosome

Target species	This study			Neavdo et al.			Shafer et al.	
		*Oncorhynchus kitush*	*Salvelinus alpinus*		*Gorilla gorilla*	Simulated	*Zalophus wollebaeki*	
Nontarget genome	*Oncorhynchus kitush*	N/A	3.64%	*Homo sapiens*	1.5%–2.5%	0.15%	*Arctocephalus gazella*	1.00%
	*Oncorhynchus tshawytscha*	1.45%	3.64%			1.00%	*Odobenus rosmarus*	2.90%
	*Oncorhynchus mykiss*	1.72%	3.42%			2.00%	*Leptonychotes weddellii*	5.10%
	*Salvelinus alpinus*	3.64%	N/A					
	*Salmo salar*	3.55%	3.22%					
	*Thymallus thymallus*	8.70%	7.81%					

### 3.2 Read mapping

**FIGURE 3:**
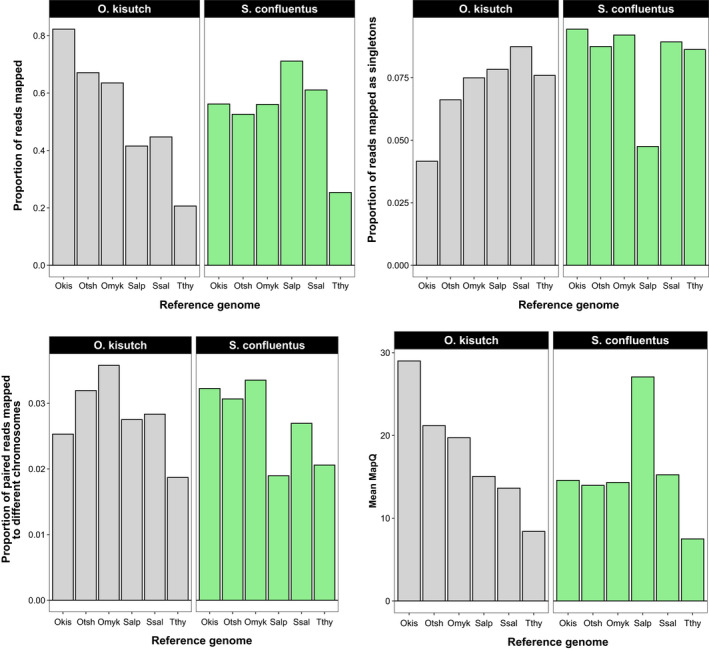
Outcome of experiment aligning RADseq data from Coho salmon (*Oncorhynchus kisutch*) and bull trout (*Salvelinus confluentus*) to different reference genomes. The reference genomes tested are on the horizontal axis and abbreviated (Okis = *O. kisutch*, Otsh = *O. tshawytscha*, Omyk = *O. mykiss*, Salp = *Salvelinus alpinus*, Ssal = *Salmo salar*, Tthy = *Thymallus thymallus*). Genomes are ordered from left to right by increasing phylogenetic distance from the Coho salmon (Okis). (a) Proportion of raw reads mapped to each genome averaged across individuals. (b) Proportion of reads mapped to each genome as singletons (i.e., without the paired read) average across individuals. (c) Proportion of reads mapped to each genome in which mates composing a set of paired reads mapped to a different chromosome. (d) Mean mapping quality scores (MapQ) averaged across individuals generated using different reference genomes

The degree of divergence between the target species and reference genome impacted alignment success. For the Coho salmon dataset, the mean proportion of reads that aligned to the reference genome was highest when the Coho salmon reference genome was used and decreased with increasing divergence from the target species (Figure 3a). On average, 80% of the reads aligned to Coho reference genome; aligning them to another species produced mean alignment rates < 70%. Aligning Coho salmon reads to non‐*Oncoryhnchus* genomes (i.e., Arctic char, Atlantic salmon, and European grayling) resulted in less than 50% of reads aligning. A similar pattern was observed with the bull trout data, with the highest proportion of reads aligning when the Arctic char genome was used as a reference. However, the proportion was still < 80%, meaning aligning bull trout data to the Arctic char genome was comparable to aligning Coho salmon data to other *Oncorhynchus* species.

The proportion of reads that mapped as singletons (i.e., only one read of a mate pair mapped) (Figure 3b) and the proportion of reads in a mate pair that aligned to different chromosomes (Figure 3c) increased as the reference genome became more diverged. MapQ for the Coho salmon RADseq alignments was highest when aligned to the Coho salmon reference genome, with a gradual decrease as the reference genome became further diverged (Figure 3d). It was highest for the bull trout data when mapped to the Arctic char genome but relatively equivalent across the other genomes.

### 3.3 Variant discovery

**FIGURE 4:**
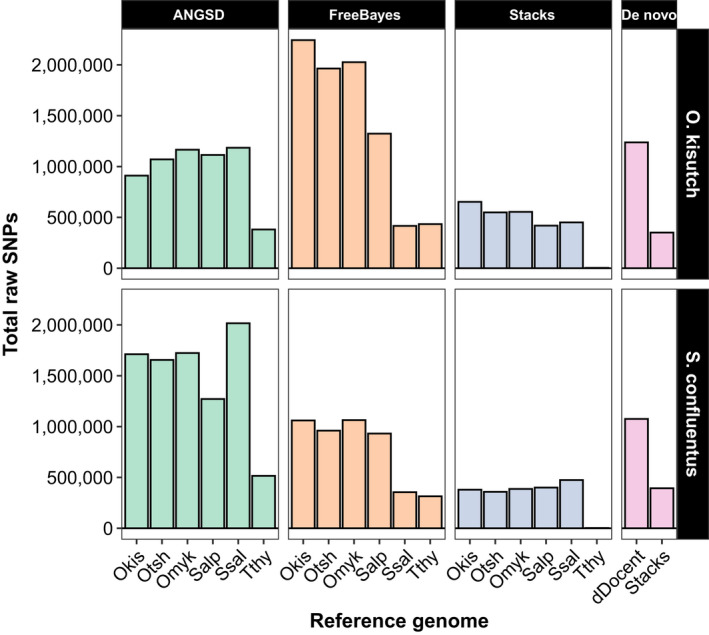
Total number of SNPs for Coho salmon (*Oncorhynchus kisutch*) and bull trout (*Salvelinus confluentus*) identified by genotyping software using different reference genomes. The reference genomes tested are on the horizontal axis and abbreviated (Okis = *O. kisutch*, Otsh = *O. tshawytscha*, Omyk = *O. mykiss*, Salp = *Salvelinus alpinus*, Ssal = *Salmo salar*, Tthy = *Thymallus thymallus*). Genomes are ordered from left to right by increasing phylogenetic distance from the Coho salmon (Okis). Runs of FreeBayes with the Atlantic salmon genome produced no useable SNPs. The right panel displays the number of SNPs identified by the two de novo pipelines

The number of SNPs generated by each of the three genotypers was highly variable for the two datasets and produced different patterns depending on the reference genome that was used to produce the alignments (Figure 4). For the Coho salmon data genotyped with ANGSD, the fewest number of raw SNPs were identified when the reads were aligned to the Coho salmon genome: More SNPs were detected as the reference genome became increasingly diverged except for the grayling genome. Similarly, the fewest SNPs detected by ANGSD with the bull trout data were when it was aligned to the Arctic char genome: other genomes produced more SNPs aside from the grayling.

With FreeBayes and Stacks, the pattern was reversed: The highest number of SNPs was generated for the Coho salmon data using the Coho salmon reference genome (Figure 4). There was a similar pattern with the bull trout data, although the highest SNP total produced by Stacks was generated with the Atlantic salmon genome. By far, the fewest SNPs were identified by Stacks: no aligner/genome combination resulted in more than 650,000 SNPs, whereas almost all FreeBayes and ANGSD analyses identified more than that regardless of reference genome.

There was a complication using FreeBayes when the Atlantic salmon genome was used as a reference. FreeBayes requested more memory than available with the computational resources I used when processing alignments from this genome, likely due to repetitive elements. As an alternative, I generated a masked genome to remove repetitive elements. After realigning the data to this masked genome, FreeBayes successfully genotyped, but produced fewer SNPs than other genomes (Figure 4).

For the de novo assembly pipelines, dDocent identified substantially more SNPs than Stacks regardless of the dataset (Figure 4). Number of SNPs identified de novo by Stacks were within the range identified by Stacks using the reference‐based alignments. The de novo dDocent pipeline uses FreeBayes to genotype: compared to when FreeBayes was used directly on the reference‐based alignments, the number of SNPs generated de novo for the Coho salmon data was much lower but comparable for the bull trout data.

**FIGURE 5:**
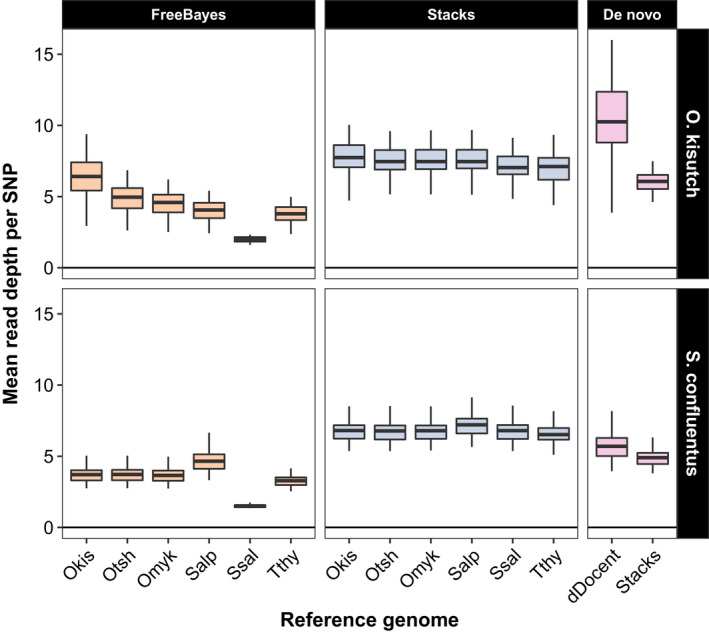
Boxplot of the distribution of mean read depth per SNP for Coho salmon (*Oncorhynchus kisutch*) and bull trout (*Salvelinus confluentus*) produced by genotyping software using different reference genomes. Depth is averaged across SNPs for each individual and based upon all raw SNPs identified by the genotypers. The reference genomes tested are on the horizontal axis and abbreviated (Okis = *O. kisutch*, Otsh = *O. tshawytscha*, Omyk = *O. mykiss*, Salp = *Salvelinus alpinus*, Ssal = *Salmo salar*, Tthy = *Thymallus thymallus*). Genomes are ordered from left to right by increasing phylogenetic distance from the Coho salmon (Okis). Runs of FreeBayes with the Atlantic salmon genome produced no useable SNPs. The right panel displays the number of SNPs identified by the two de novo pipelines

Despite the large discrepancies in the number of raw SNPs that were identified, read depth at those SNPs averaged across individuals was not substantially different between FreeBayes and Stacks (Figure 5). Comparatively, FreeBayes had lower mean depth across all identified SNPs and was impacted more by reference genome divergence. SNPs produced by FreeBayes had higher coverage with decreasing divergence between the target species and reference genome (i.e., Coho salmon data aligned to Coho salmon genome, bull trout data aligned to Arctic char genome). Stacks was relatively consistent in mean depth per SNP: There was a slight trend in lower depth with increasing reference genome divergence but it was not substantial. SNPs identified de novo by Stacks had lower coverage than those identified with reference genomes; the opposite was true with dDocent with de novo SNPs having higher coverage than the FreeBayes reference‐generated SNPs.

### 3.4 Population genetics

**FIGURE 6:**
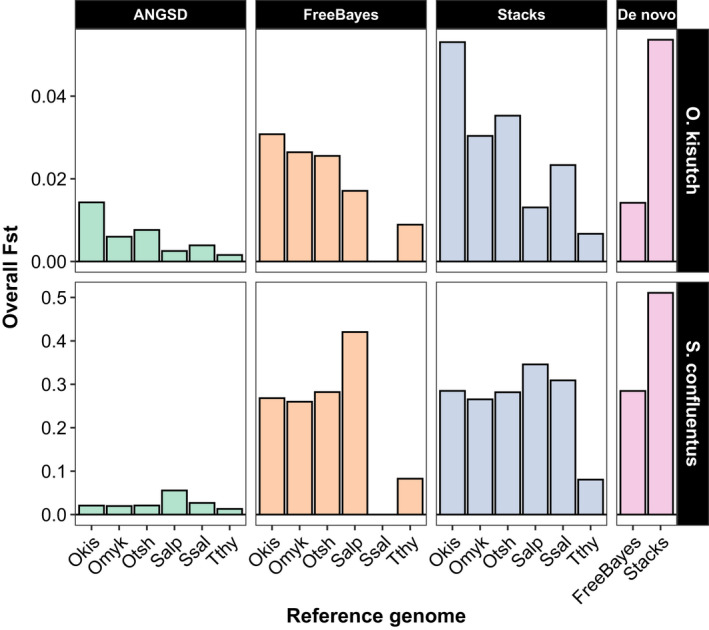
Global estimates of *F*_ST_ for both the Coho salmon (*Oncorhynchus kisutch*) and bull trout (*Salvelinus confluentus*) RADseq datasets aligned to different reference genomes. The reference genomes tested are on the horizontal axis and abbreviated (Okis = *O. kisutch*, Otsh = *O. tshawytscha*, Omyk = *O. mykiss*, Salp = *Salvelinus alpinus*, Ssal = *Salmo salar*, Tthy = *Thymallus thymallus*). Genomes are ordered from left to right by increasing phylogenetic distance from the Coho salmon (Okis). The estimates are also grouped by the genotyping software that was used to produce the estimates. Runs of FreeBayes with the Atlantic salmon genome produced no useable SNPs

Several trends were noticeable with the estimates of *F*_ST_. First, there were differences in the magnitude of values depending on the genotyper. In general, FreeBayes and Stacks produced estimates within a similar range; ANGSD consistently produced much lower estimates, especially for the bull trout data (Figure 6). Second, regardless of genotyper or species, there was a tendency for lower estimates of *F*_ST_ with increasing genome divergence. Estimates with the Coho salmon data showed a gradual decline with genome divergence; with the bull trout, *F*_ST_ was highest when the data were aligned to the Arctic char genome and comparable when other genomes were used. The two de novo pipelines had opposite patterns: The dDocent pipeline produced lower *F*_ST_ estimates than when FreeBayes was run on the reference‐aligned data. In contrast, de novo Stacks datasets produced the highest estimates of *F*_ST_, higher than with the reference‐aligned Stacks data. Confidence intervals bounded *F*_ST_ estimates tended to be narrow (Table 3) and overlap between estimates generated with the same dataset was variable. Due to using the masked genome, SNPs generated by FreeBayes using the Atlantic salmon genome produced too few SNPs following filtering to be used in the population genetic analyses.

**TABLE 3:** *F_ST_* values generated for A. Coho salmon (*Oncorhynchus kisutch*) and B. Bull trout (*Salvelinus confluentus*) RADseq datasets using different reference genomes and genotypers. Provided are the 95% confidence intervals (in parentheses) for the point estimates. The reference genomes tested are abbreviated (Okis = *O. kisutch*, Otsh = *O. tshawytscha*, Omyk = *O. mykiss*, Salp = *Salvelinus alpinus*, Ssal = *Salmo salar*, Tthy = *Thymallus thymallus*). Genomes are ordered from top to bottom by increasing phylogenetic distance from the Coho salmon (Okis). De novo refers to the assembly pipelines of Stacks and dDocent, which uses FreeBayes to genotype. The Atlantic salmon genome (Ssal) processed with FreeBayes produced no useable SNPs

(A) Genome	ANGSD	FreeBayes	Stacks	De novo
*Oncorhynchus kitush*	0.014 (0.014–0.015)	0.031 (0.029–0.033)	0.053 (0.049–0.057)	
*Oncorhynchus tshawytscha*	0.008 (0.007–0.008)	0.026 (0.023–0.028)	0.035 (0.032–0.039)	
*Oncorhynchus mykiss*	0.006 (0.006–0.006)	0.026 (0.023–0.029)	0.03 (0.027–0.034)	
*Salvelinus alpinus*	0.003 (0.002–0.003)	0.017 (0.014–0.02)	0.013 (0.011–0.015)	
*Salmo salar*	0.004 (0.003–0.004)	NA	0.023 (0.02–0.026)	
*Thymallus thymallus*	0.002 (0.001–0.002)	0.01 (0.01–0.01)	0.007 (0–0.016)	
FreeBayes				0.01 (0.01–0.02)
Stacks				0.05 (0.05–0.06)

(B) Genome	ANGSD	FreeBayes	Stacks	De novo
*Oncorhynchus kitush*	0.02 (0.02–0.02)	0.27 (0.26–0.28)	0.28 (0.27–0.3)	
*Oncorhynchus tshawytscha*	0.02 (0.02–0.02)	0.28 (0.27–0.3)	0.28 (0.27–0.29)	
*Oncorhynchus mykiss*	0.02 (0.02–0.02)	0.26 (0.25–0.27)	0.27 (0.25–0.28)	
*Salvelinus alpinus*	0.06 (0.05–0.06)	0.42 (0.41–0.43)	0.35 (0.34–0.35)	
*Salmo salar*	0.03 (0.03–0.03)	NA	0.31 (0.3–0.32)	
*Thymallus thymallus*	0.01 (0.01–0.01)	0.08 (0.07–0.09)	0.08 (0.04–0.12)	
FreeBayes				0.28 (0.28–0.29)
Stacks				0.51 (0.5–0.52)

**FIGURE 7:**
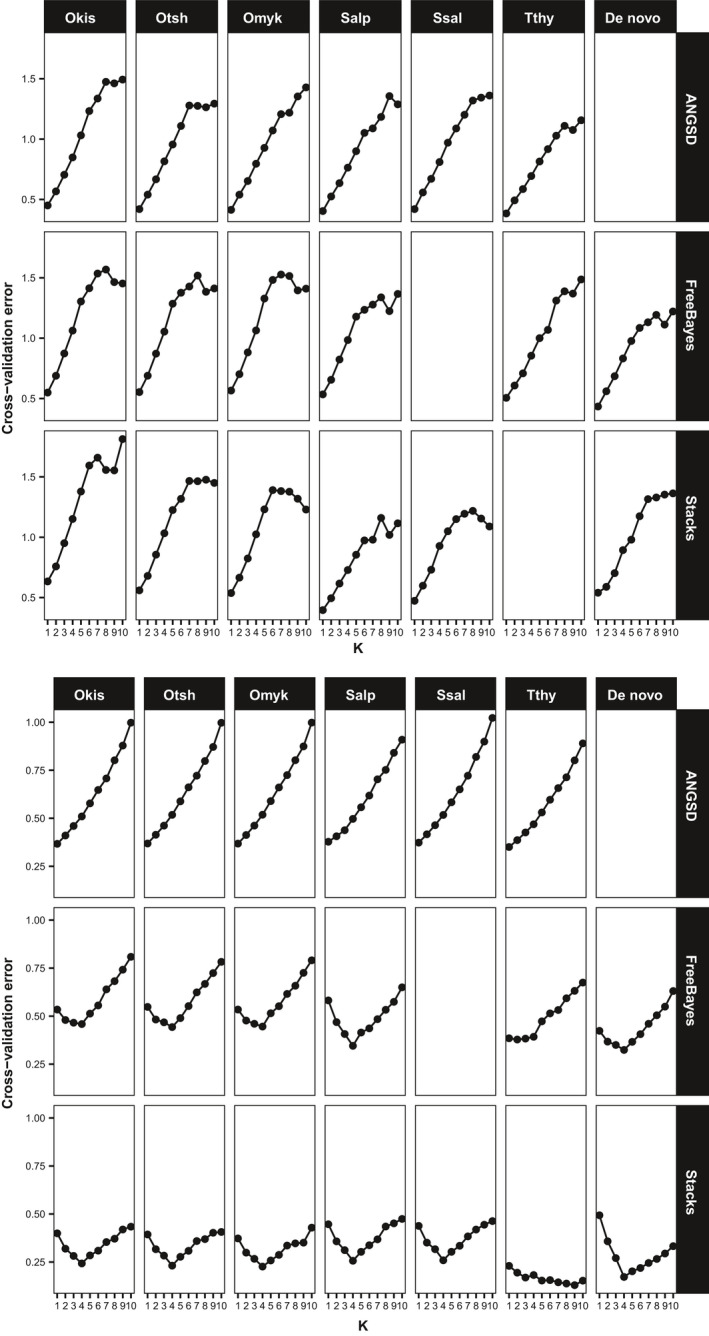
Cross‐validation error across *K* values varying from 1 to 10 for the *admixture* clustering analysis of the RADseq datasets. Panel A is for the Coho salmon (*Oncorhynchus kisutch*) and Panel B for the bull trout (*Salvelinus confluentus*). The lowest error value is often interpreted as the most feasible clustering patterns among the data. The reference genomes tested are on the horizontal axis and abbreviated (Okis = *O. kisutch*, Otsh = *O. tshawytscha*, Omyk = *O. mykiss*, Salp = *Salvelinus alpinus*, Ssal = *Salmo salar*, Tthy = *Thymallus thymallus*). Genomes are ordered from left to right by increasing phylogenetic distance from the Coho salmon (Okis). Runs of FreeBayes with the Atlantic salmon genome produced no useable SNPs. For the Coho salmon data when Stacks was used with the grayling genome (Tthy) it produced a low number of SNPs and the *admixture* run failed

The cross‐validation error rates produced by *admixture* were comparable regardless of genotyper or reference genome (Figure 7). Every single Coho salmon dataset run with *admixture* had the lowest error at *K* = 1 with a steady increase in error at high values. For the bull trout, with Stacks and FreeBayes the lowest error was at *K* = 4 regardless of reference genome except for the data aligned to the grayling genome. The bull trout datasets produced by ANGSD had the lowest error at *K* = 1 regardless of reference genome.

**FIGURE 8:**
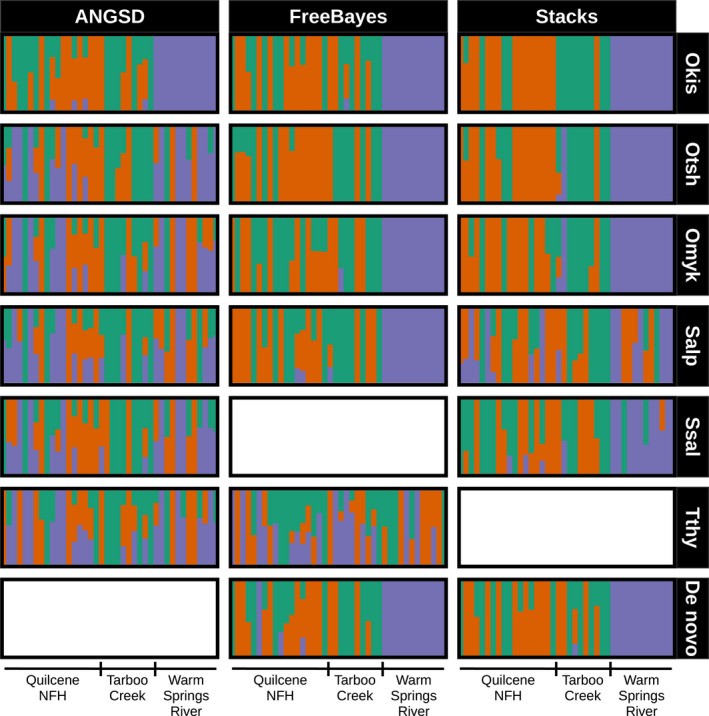
Clustering patterns for the Coho salmon (*Oncorhynchus kisutch*) RADseq datasets aligned to different reference genomes. Barplots represent individual runs of the dataset with different genome and genotyper combinations. These plots are based on running *admixture* assuming three clusters. Each cluster is represented by a different color. The horizontal bars at the bottom of the plot denote the population of origin of the individual Coho salmon. The reference genomes tested are on the horizontal axis and abbreviated (Okis = *O. kisutch*, Otsh = *O. tshawytscha*, Omyk = *O. mykiss*, Salp = *Salvelinus alpinus*, Ssal = *Salmo salar*, Tthy = *Thymallus thymallus*). Genomes are ordered from left to right by increasing phylogenetic distance from the Coho salmon (Okis). Runs of FreeBayes with the Atlantic salmon genome produced no useable SNPs. For the Coho salmon data when Stacks was used with the grayling genome (Tthy) it produced a low number of SNPs and the *admixture* run failed

I compared clustering patterns at *K* = 3 for the Coho salmon data and *K* = 4 for the bull trout data, for these were the number of known populations that were included in each respective dataset. The Warm Springs River was expected to be more divergent from the other two populations, but it did not form a distinct cluster with every genome/genotyper combination (Figure 8). With ANGSD, it was only distinct when the Coho genome was used: no other genomes produced any clear clustering patterns. In contrast, there was a clear Warm Springs cluster when SNPs were produced by FreeBayes using all the genomes except for the grayling. It only formed a distinct cluster with Stacks when *Oncorhynchus* genomes were used. The dataset produced by Stacks with the grayling genome had so few SNPs that the *admixture* run failed. Both de novo assemblies resulted in a distinct Warm Springs cluster. No combination clearly separated the Quilcene NFH and Tarboo Creek populations: although two clusters were identified among these individuals, they did not clear correspond to the two populations. Admixture proportions also variety across genome/genotyper combinations.

**FIGURE 9:**
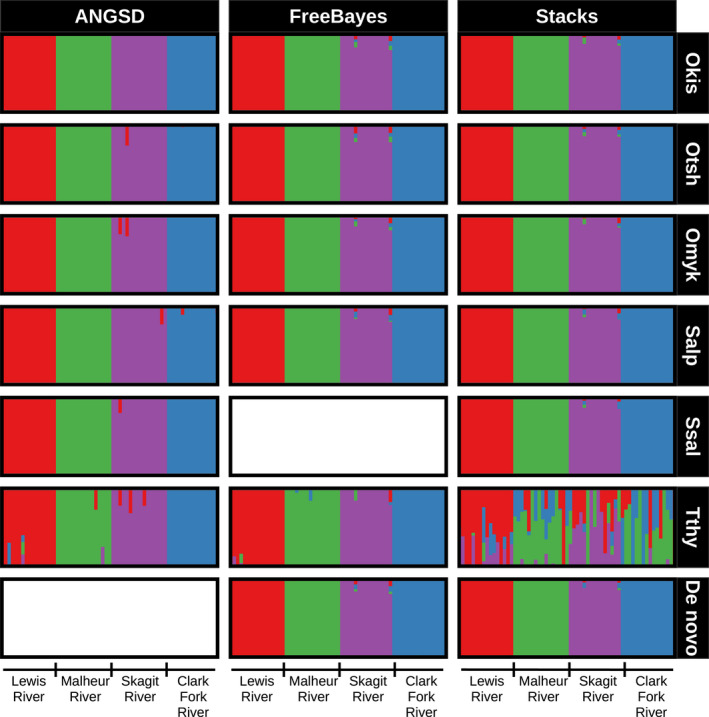
Clustering patterns for the bull trout (*Salvelinus confluentus*) RADseq datasets aligned to different reference genomes. Barplots represent individual runs of the dataset with different genome and genotyper combinations. These plots are based on running *admixture* assuming four clusters. Each cluster is represented by a different color. The horizontal bars at the bottom of the plot denote the population of origin of the individual bull trout. The reference genomes tested are on the horizontal axis and abbreviated (Okis = *O. kisutch*, Otsh = *O. tshawytscha*, Omyk = *O. mykiss*, Salp = *Salvelinus alpinus*, Ssal = *Salmo salar*, Tthy = *Thymallus thymallus*). Genomes are ordered from left to right by increasing phylogenetic distance from the Coho salmon (Okis). Runs of FreeBayes with the Atlantic salmon genome produced no useable SNPs

The bull trout clustering at *K* = 4 was consistent every time: every single genome/genotyper combination resulted in the same clustering pattern with the exception of the Stacks data produced with the grayling genome (Figure 9). These clusters corresponded to the four known populations.

## 4 DISCUSSION

### 4.1 Effect of genome divergence

The practice of aligning HTS sequences generated for one species to the genome of another has been a necessary evil for biologists due to the logistical constraints of producing reference genomes for every species on Earth. However, this study demonstrates that the reference genome is an important component of variant discovery. Substantial space has been given in the literature to assessing the impacts of various aspects of bioinformatics pipelines, including alignment and genotyping software, de novo versus reference‐aligned, and data filtering (Andrews, Good, Miller, Luikart, & Hohenlohe, 2016; Fountain, Pauli, Reid, Palsbøll, & Peery, 2016; Hodel et al., 2017; Mastretta‐Yanes et al., 2015; Nielsen et al., 2011; Paris et al., 2017; Shafer et al., 2017). The choice of reference genome should also be added to the list of variables geneticists should carefully evaluate when designing a study.

Perhaps unsurprisingly, greater divergence between the target species and reference genome resulted in fewer mapped reads and lower mapping quality. Although other studies have shown that using nontarget reference genomes affect SNP calling and downstream analysis (Gopalakrishnan et al., 2017; Nevado et al., 2014; Shafer et al., 2017), they have not documented whether this extended to the initial phase of read mapping as well. Results from this study suggest the impacts of reference genome divergence are rooted in the alignment process, which would subsequently affect downstream analyses. Parameters could be adjusted during the alignment process to account for this, but it will not eliminate the problem. RADseq data may be particularly sensitive to reference genome divergence given that mutations in the restriction cut‐site would lead to complete absence of overlapping sequence data between species (Arnold, Corbett‐Detig, Hartl, & Bomblies, 2013; Davey et al., 2013). Salmonids add another wrinkle in that the ancestor to the entire family underwent a whole‐genome duplication around 65 million years ago event that was subsequently rediplodized (Varadharajan et al., 2017). The presence of paralogous regions of the genome may pose problems for read mapping, further exasperating the impact of using a divergent reference genome.

The most practical consequence of this phenomenon is that perfectly good sequencing reads may be removed from a dataset simply due to the choice of reference genome, reducing overall genome coverage. Another issue is that the reads that do map do so with less precision as the reference genome becomes more diverged, evidenced by mate pairs mapping to different chromosomes and the MapQ scores. For genotyping algorithms that incorporate these metrics into variant discovery, as both FreeBayes and ANGSD do, this may result in fewer and more error‐prone variants.

Although mapping metrics and SNP discovery matter, at the end of the day for many practical applications, the major issue is whether reference genome divergence impacts biological inference. To this point, the results from this study were mixed. Clearly, estimates of *F*_ST_ were impacted: Populations appeared less differentiated as the reference genome became more divergent. However, clustering patterns produced by admixture were highly consistent for the bull trout data, but less so for the Coho salmon. This could be related to the properties of the two analyses: *F*_ST_ is computed using the mean allele frequencies across populations. Less data in the form of fewer aligned reads could lead to less accurate allele frequency estimates. Furthermore, if identified SNPs reflect fixed differences between the target species and reference genome, a large portion of the allele frequencies may be homogenous across populations. In contrast, clustering algorithms identify patterns based on Hardy–Weinberg discontinuities across individuals: Even if individuals share the same genotypes across large numbers of SNPs, consistent differences in genotype frequencies at several loci can be detectable.

It is also notable that the clustering results were more consistent in detecting deeper divergences between populations. The bull trout populations, which originate from highly diverged phylogenetic lineages, were consistently differentiated. However, Coho salmon lineages across the West Coast are not particularly well‐defined, which would explain why the Warm Springs River population did not always cluster separately from the two Hood Canal populations. The Quilcene NFH and Tarboo Creek populations were never identified as separate clusters. Broodstock propagated at Quilcene NFH were founded from local populations within the past several decades, meaning allele frequencies may not have significantly diverged to be detected by *admixture*. Alternatively, the observed mixing of the two populations may be a real biological signal: Tarboo Creek and Quilcene NFH are within close proximity, and migrants have been found between the two populations (Bohling et al., 2020). However, considering that admixture proportions varied for individuals across genome/genotyper combinations suggests the shallow divergence played a large role in the lack of clear clustering. Plus, Bohling et al. (2020) found the broodlines propagated at Quilcene NFH were distinct from naturally spawning local populations. Overall, using more divergent reference genomes for alignment may be practical when detecting deep patterns of population structure, but may cause problems in detecting more recent population splits.

### 4.2 Genotypers

There were also differences in how the three genotyping software programs responded to reference genome divergence, making general characterizations difficult. By far, more SNPs were produced by FreeBayes and ANGSD relative to Stacks. This makes sense as FreeBayes and ANGSD are more amenable to low coverage data and incorporate Bayesian models that use the full suite of sequencing data to identify variants. However, Stacks was much less impacted by reference genome divergence. Stacks does not depend on the reference genome for actually calling SNPs: It identifies variants within individuals based on base calls and then combines those across individuals. Both FreeBayes and ANGSD will call SNPs based on base call differences between the HTS reads and the reference genome itself, explaining why reference genome divergence did not decrease the number of overall SNPs, especially for ANGSD. This could create a false impression of data robustness, generating a large number of SNPs that are totally homozygous within the target populations (Nevado et al., 2014). Genotyping approaches like those implemented by Stacks may be a safer choice when HTS data are aligned to a divergent, nontarget reference genome.

Inferences derived with SNPs identified by ANGSD diverged considerably from the other two genotypers. One contributing factor may have been the choice of analytical pipelines: providing a fair comparison between the genotypers meant using the same analysis software, which meant actually calling genotypes with ANGSD. The strength of ANGSD, however, it is calculation of genotype probabilities instead of called genotypes (Waples, Albrechtsen, & Moltke, 2019; Warmuth & Ellegren, 2019). This approach has several advantages, especially with low coverage data, and can provide accurate estimates of allele frequencies (Korneliussen et al., 2014; Nielsen, Korneliussen, Albrechtsen, Li, & Wang, 2012). The problem is that many of the traditional software applications in population genetics are not designed for genotype probabilities: Specialized software must be used to exploit this output (Fumagalli, Vieira, Linderoth, & Nielsen, 2014; Skotte, Korneliussen, & Albrechtsen, 2013). Thus, providing a direct comparison between called genotypes and probabilities is problematic. For example, ngsAdmix, the equivalent of *admixture* that was developed to perform clustering of ANGSD output, does not perform the cross‐validation procedure for inferring *K*. Nor is there a straightforward way to calculate overall *F*_ST_ among more than two populations using genotype probabilities. Using genotype probabilities has inherent appeal with HTS data, but software applications must be developed to meet the diverse needs of geneticists for it to reach its full potential.

### 4.3 Implications for biodiversity genomics

There are several additional observations gleaned from this study that serve as important lessons for geneticists as we advanced into the genomic era. First relates to the de novo assemblies compared to the reference‐aligned datasets. Several studies have suggested for RADseq data that higher coverage and genotyping accuracy are achieved with reference‐aligned pipelines compared to de novo (Fountain et al., 2016; Shafer et al., 2017; Torkamaneh, Laroche, & Belzile, 2016). Analyzing these salmonid datasets suggests that the question of de novo versus reference‐aligned is more nuanced. Whether the de novo pipelines produced fewer SNPs depended on the dataset: with bull trout RADseq data, the de novo numbers were comparable to reference aligned. Mean read depth at SNPs was higher for the dDocent de novo pipeline than with the FreeBayes reference‐aligned data, but the opposite was true with Stacks. The highest estimates of *F*_ST_ were obtained with data assembled de novo by Stacks, suggesting it was emphasizing divisions between our populations. Paris et al. (2017) suggested that de novo assemblies take full advantage of the information contained within RADseq data, some of which may be lost due to poor alignment or difference between the target species and reference genome. Thus, de novo assemblies may be the preferred approach in situations when there is no genome from the target species or any closely related species.

A final observation concerns the varying estimates of population differentiation generated by all the genome/genotyper combinations. Even though produced with the same underlying sequence data, estimates of *F*_ST_ and clustering patterns were variable. These findings echo those of Nevado et al. (2014), Gopalakrishnan et al. (2017), and Shafer et al. (2017) and emphasize an important point: biological inferences can be unpredictable when nontarget species reference genomes are used. As researchers have examined the factors that influence genotyping with HTS, a common thread has emerged that changing a few parameters can alter population genetic estimates (Fountain et al., 2016; Hodel et al., 2017; Mastretta‐Yanes et al., 2015; Paris et al., 2017). Even the choice of software should be viewed as a parameter that can influence biological inference (Nevado et al., 2014; Shafer et al., 2017; Torkamaneh et al., 2016).

This has profound consequence for interpretation, especially in situations in which findings have consequences for conservation and other applied applications. Geneticists should be transparent that processing a single dataset through a single pipeline represents a single snapshot view of biodiversity. Researchers should be careful in assuming inferences made when aligning HTS data to the genome of a nontarget species will hold up when another genome is used. Unless consensus builds that particular pipelines and parameters are required for genomic studies, it would be advisable to test varying parameters and software to gain a more generalized view of biological patterns.

To close, this study reveals that the reference genome is an important variable in the processing of HTS data. Over the past decade, there have been a variety of initiatives to develop reference genomes for as many species as possible, such as the Genome 10K project (Genome 10K Community of Scientists, 2009; Koepfli, Paten, Genome 10K Community of Scientists, & O’Brien, 2015) and Earth BioGenome Project (Lewin et al., 2018). One practical application of these initiatives is that reference genomes will be available for conducting intraspecific population analyses, reducing the need to align HTS data to more diverged genomes. This would increase the veracity of studies using HTS data, resulting in better coverage and higher‐quality variants. It is tempting to generate HTS data to investigate a wide array questions we have in genetics: however, we may need to assess whether we are putting the cart before the horse in generating these data before reference genomes are available for these species.

## CONFLICT OF INTEREST

The author declares that there are no conflicts of interest.

## AUTHOR CONTRIBUTION

**Justin Bohling conceived and designed the study, performed the data analysis and interpretation, and wrote and edited the article.**

## Supporting information

Supplementary Material

## Data Availability

The genolames used for this study are publically available. The RADseq data have been described in previous publications (Coho data, https://doi.org/10.1002/tafs.10206; bull trout data, https://doi.org/10.1007/s10592‐018‐1134‐z) that include instructions for data accessibility. All software used for the data analysis is open source, and the specific scripts used for the bioinformatics processing are provided in the Supplemental Material.
